# The Cranial Bowl in the New Millennium and Sutherland's Legacy for Osteopathic Medicine: Part 2

**DOI:** 10.7759/cureus.10435

**Published:** 2020-09-14

**Authors:** Bruno Bordoni, Stevan Walkowski, Bruno Ducoux, Filippo Tobbi

**Affiliations:** 1 Physical Medicine and Rehabilitation, Foundation Don Carlo Gnocchi, Milan, ITA; 2 Osteopathic Manipulative Medicine, Heritage College of Osteopathic Medicine-Dublin, Dublin, USA; 3 Osteopathy, Formation Recherche Osteopathie Prévention, Bordeaux, FRA; 4 Osteopathy, Poliambulatorio Medico e Odontoiatrico, Varese, ITA

**Keywords:** osteopathic, cranio, fascia, craniosacral, manual therapy

## Abstract

Cranial osteopathic medicine is practiced all over the world, respecting the dictates of the creator, Dr Sutherland. Despite the current manual approach faithfully follows the theoretical and practical bases that make up the cranial model of the last century, there are many scientific evidences that highlight the criticalities of the same model. In the first part we reviewed the role of the meninges and cerebrospinal fluid (CSF), as well as we discussed some rhythms present in the central nervous system; these latter elements are the pillars to support the theoretical idea of the movement of the skull evaluated and palpated by the osteopath. In this second part we will review the mechanical characteristics of other structures that make up the cranial system, highlighting new perspectives for clinical practice, thanks to the most recent data derived from scientific research.

## Introduction and background

Osteopathic cranial manipulative medicine (OCMM) was born from the intuitions of a student of Dr Still (founder of osteopathic medicine), Dr Sutherland [1]. The logic of OCMM is based on the primary respiratory mechanism (PRM), a theoretical model built with five principles, to illustrate the reasons for cranial movement or cranial rhythm in the osteopathic field: the fluctuation of the cerebrospinal fluid (CSF); the inherent motility of the central nervous system and spinal cord; the mobility of the meningeal membranes (cranial and spinal); the joint mobility of the bones of the skull; and the involuntary (passive) movement of the sacrum between the iliac bones [1]. Cranial manipulation was introduced as a clinical practice of the osteopathic physician in 1930, while it became a subject of university study in 1940 [2]. The evaluation and manual work on the skull does not mean that the rest of the body is not taken into consideration, as osteopathy considers the patient a unit with always interdependent anatomical relationships [3]. According to Dr Viola Frymann, palpation of the rhythm of the skull is feasible and does not depend on the pulsation of the arterial vessels that feed the brain; the Traube-Hering rhythm, often associated with the rhythm of the skull, does not actually represent the rhythm palpated by the osteopath [4]. The values recorded by Dr Traube and Dr Hering were derived from animal experiments with the thorax open and the diaphragm muscle paralyzed [4]. The cranial rhythm can also be palpated by placing the hands on the rest of the body, creating a dichotomy with the Traube-Hering waves [5]. We must also consider that human palpation is very fine and capable of recognizing very small objects, measuring 10 nm or 0.01 μm [6]. This ability is within the range of the movement of the cranial sutures. For some scholars, the sutures of the skull in adults, being still recognizable, do not merge but only become very adherent; in addition, the sutures between the bones that form the skull may have movements of the order of 5000-1700 nm or 5-17 microns, palpable by humans [5]. According to the osteopathic view, each bone that makes up the skull moves with its own axes and planes, making the sutures a joint; the movement patterns have been created artificially and with a theoretical basis to obtain reference points during palpation [7]. Sutherland himself asserts that such schemes do not necessarily correspond to reality [8]. The joint that represents the engine of cranial movement and to which the various dysfunctions devised by Sutherland are attributed, is the synchondrosis between the occipital bone and the sphenoid bone, the sphenobasilar synchondrosis (SBS), both in adults and children [7]. The second part of the article will review the SBS and the sutural function of the skull, the mechanical characteristics of the brain, the movement of the sacral bone, reflecting on the need for a new osteopathic cranial model. The article, as specified in the first part, takes into consideration the scientific information of the adult skull, leaving out the skull of the child and the elderly.

## Review

Joint mobility of the skull bones

**Figure 1:**
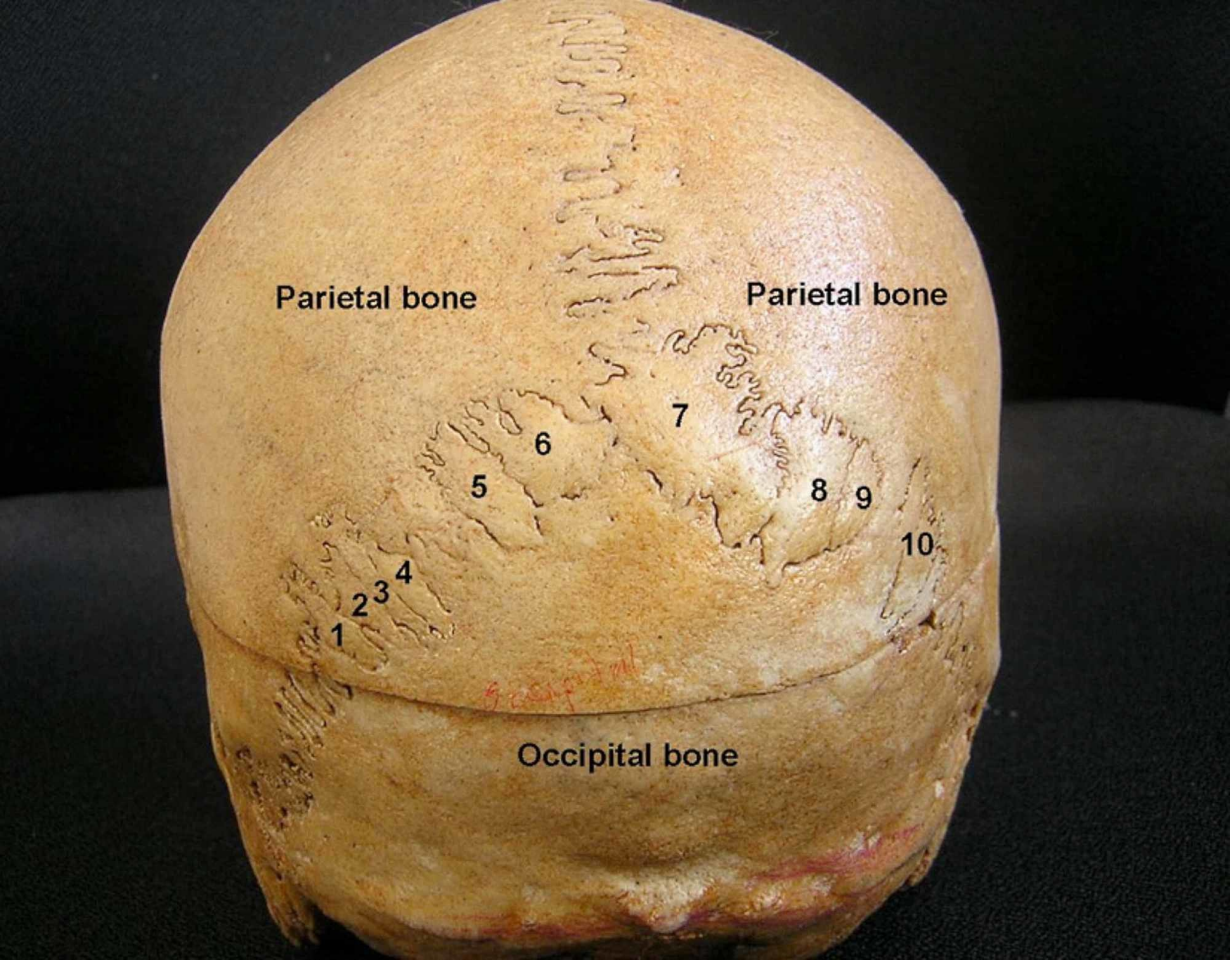
The posterior view of the skull showing 10 sutural bones (Wormian bones). Thanks to Dr Satheesha Nayak B, associate professor of Anatomy, Melaka Manipal Medical College (Manipal Campus), International Centre for Health Sciences, Madhav Nagar, Manipal, Udupi District, Karnataka State, India.

The adult skull is made up of 29 bones, in general, but we do not always know exactly how many sutures there are (about 15). The literature states that the size of the skull depends on the growth of the brain, while the sutures determine the shape [9]. On the surface of the bone itself, for example the parietal bone, we can find sutures that do not correspond to the classic anatomical view; sutures in greater numbers create independent bones (Wormian bones or Inca bones), such as pre-interparietal and interparietal bones, or single bones found on one side or more bones on the major suture (lamboid suture) [9]. The bones and sutures in greater numbers would depend on the ossification centers that are not always taken into account [10]. The Wormian bones and, consequently, of the nonlinear joint relationships, would be found mainly on the right area of the skull and for about 50% would involve the lambdoid suture; 25% would involve the coronal suture, while the rest of the Wormian bones are found in different areas of the skull [10]. Depending on the population, Wormian bones would be found with a percentage of 8%-15% in Western civilizations; in the Chinese population, the excess bones would be present in 80% of the population [10]. There is no agreement whether there is differences between sexes [10]. If there is the presence of more bones, this creates a possible pathological clinical picture (pyknodysostosis, rickets, osteogenesis imperfecta, and others) [9-10]. There may be other types of sutures in the adult skull, such as mendosal sutures; the latter are very short (from 0.8 mm to 1.4 cm), and arise from the medial portion of the lambdoid suture, towards the occipital bone [11]. The evaluative palpation of the skull (and vertebrae) by the osteopath should have two objectives: to check the position and mobility [12]. But what is the origin of the cranial rhythm described by Sutherland in which the bones of the skull move? These are fundamental questions to understand the scientific reality of osteopathic work. In the first part of the article we demonstrated how the membranes are able to transmit the movements of the brain induced by the heartbeat and diaphragmatic breathing, slowing down the frequency, thanks to the intrinsic meningeal characteristics. The sutures of the skull or synarthroses, albeit with different characteristics, are able to decrease the frequency of the mechanical stimuli that reach them [13]. The higher the frequency of the mechanical stimulus, the lower the magnitude of the distributed stress [13]. This feature coincides with our observation of the secondary respiratory mechanism (SRM), where the rhythms of the skull have depended on cardiac and respiratory activity, although the rhythm palpated by the osteopath is slower. The morphologically more complex sutures are able to handle greater mechanical force [14]. The sutures of the skull are made up of fibrous tissue with two layers of osteoblasts, the last layers of which contain mesenchymal cells; the upper layer is covered by the periosteum, while the lower layer is in contact with the dura mater [15]. In the space of mesenchymal cells we find the nerve growth factor (NGF), which allows the nerve present in the suture (trigeminal nerve from the dura mater), to survive and function properly [1,16]. These nerve fibers are proprioceptive but, in case of injury, they can become nociceptive [16]. Furthermore, the present nervous tissue is capable of repairing the tissue in the event of a fracture; the presence of mesenchymal cells, and the presence of nervous tissue capable of stimulating the reparative processes in a flattened suture of the skull, may lead us to hypothesize that the cranial sutures are active and participate in the mechanometabolic stimuli of the body. When there is a structure in the human body, it is because the same structure is used and has one or more purposes or function. The craniofacial sutures remain partially open in the adult stage (they do not ossify), and in the elderly [1]. Sutures such as the occipito-mastoid and the parieto-mastoid remain nonossified until the age of 80; the spheno-parietal and spheno-frontal remain nonossified until the age of 60 (Figure 1) [1].

There are studies showing that the bones of the skull can move under physiological stimuli, with an average of about 17-70 microns (higher values than previous reviews) [5, 17-18]. Probably, not only the sutures allow the transmission of forces deriving from the movement of the nervous system but also the structure of the skull bone itself. The bones that make up the skull have elastic properties, with the temporal bone having the maximum ability to withstand tension, down to the least compliant bone such as the frontal bone [19]. Furthermore, the underlying dura mater and the periosteum, which is a continuum with the underlying tissue through the Sharpey's perforating fibers, collaborate for the most correct management of mechanical signals [19]. The bone tissue contains elastin and collagen and together they ensure a capacity of mechanical deformation of about 10%-15%. The skull bone behaves differently than the bones of the body. If the bones of the human body lose their elastic capacity with age, the bones of the skull do not undergo this adaptation [20]. The intrinsic structure of the cranial bones, like the bones of the body, contain osteocytes, but the latter behave differently. The osteocyte that derives from the osteoblast, constitutes about 90% of the bone, is soaked in the bone matrix, and represents the needle of the balance of the health of the tissue itself; they constitute a network of small channels (such as dendritic cells), with which they communicate with other osteocytes, osteoblasts and osteoclasts and all the fluids contained in the bone [21]. If the number decreases, as happens with old age, the bone loses its plastic and elastic qualities; this event does not happen in the bones of the skull, including the mandible [21]. Bone fluids (blood and water) are equally important for a physiological distribution of mechanical signals between the outside and inside of the bone; the fluids cause the osteocyte to vibrate through metabolic alterations (calcium variations), thus allowing optimal tissue adaptation to be obtained [22]. The lower the mechanical force that reaches the bone (maximum 10 Hz), the better the fluid response and the management of tension [23]. Another aspect of OCMM is the attribution of axes and planes of movement to the individual bones, which the palpatory evaluation of the osteopath must take into account [2]. Based on these axes and planes, cranial dysfunction is described, and ultimately should be the focus of osteopathic clinical treatment, either between the joint relationships between two bones or in the general context of the whole skull [2]. We know that sutures and cranial bones can be found in greater numbers and we know that the space occupied by the sutures inside the skull is different, compared to the external palpated space [24]. The sutural joints, synarthrosis or synchondrosis, dentate or squamous, do not perform rotational or flexion-extension movements; if we want osteopathic medicine to be considered equal with other scientific disciplines, we will have to step back and reconsider the movement between the bones of the skull with new perspectives.

Sphenobasilar synchondrosis

**Figure 2:**
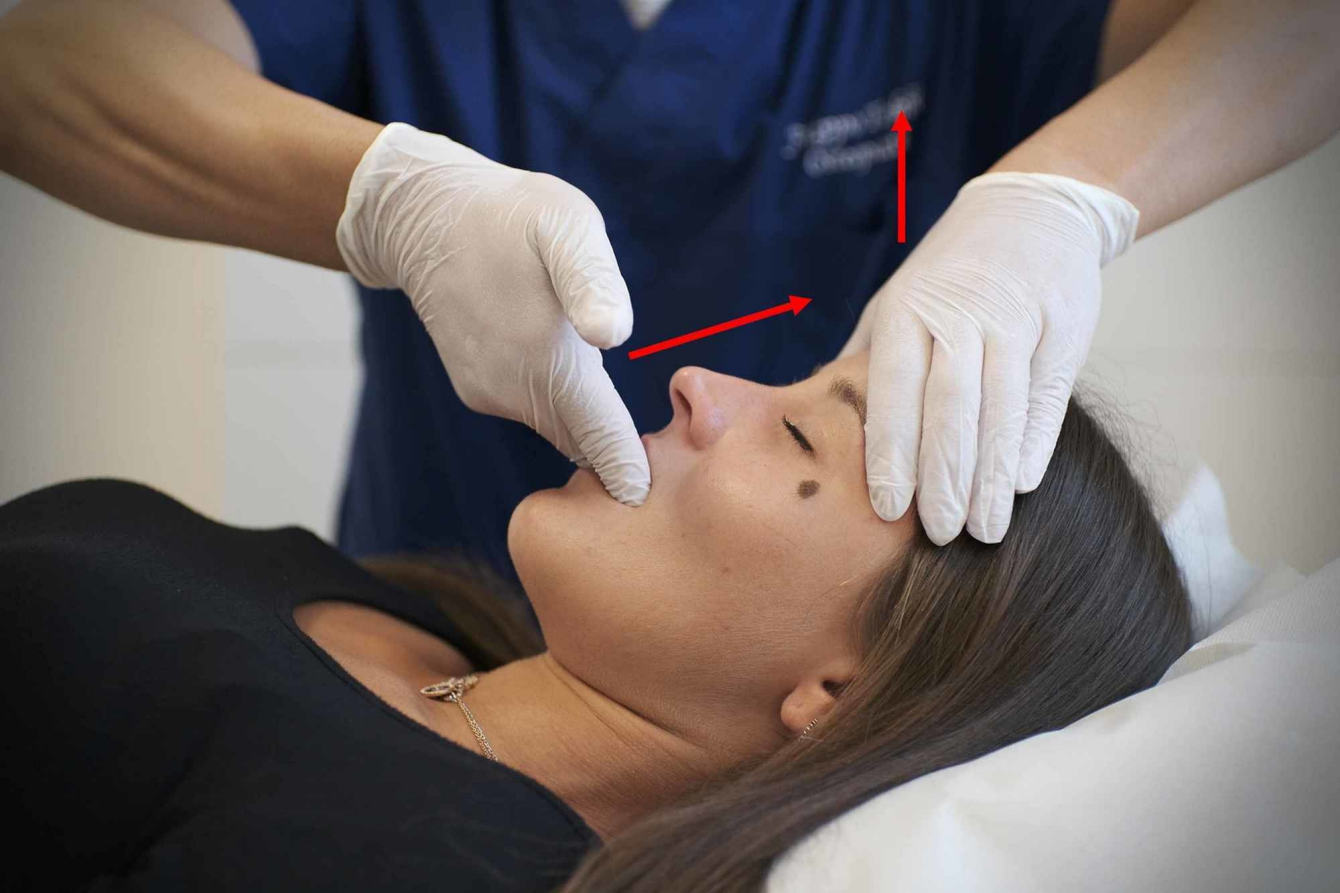
Manual decompression of the sphenobasylar joint, this technique is of no value in the light of current scientific information. The intrabuccal fingers push the upper dental arch in a cranial direction, while the other hand tries to give a traction from the pillars of the frontal bone, towards the ceiling. The figure is owned by Prof. Bordoni Bruno, the technique is performed by Dr Tobbi Filippo with a colleague in a supine position. The technique can be found cited in Ref. [2].

According to OCMM, the fulcrum that allows the bones to move with patterns and axes is the SBS [2]. If the joint relationship between the base of the occiput and the sphenoid body shows a dysfunction, the latter will be responsible for specific positional anomalies of the bones of the skull, detectable by osteopathic palpation. In fact, the movement patterns described in osteopathic medical texts may be the result of tactile illusions induced by the same study, as Dr Frymann wrote [4]. This reflection does not question the fact that the osteopath's hands are trained on palpatory listening, is able to perceive very small changes in the skull [25]. We know that the SBS begins to undergo an ossification process before puberty, with an intracranial departure, to end within the pubertal cycle [1]. The adult skull has an ossified SBS and, from a scientific point of view, it is not possible to think of this joint as the principle of cranial movement or as the cause of the various dysfunctions described in OCMM [1-2]. Some manual approaches that aim to free this ossified joint must be reconsidered (Figure 2).

Mechanical properties of the brain

To try to fully understand the behavior of the skull from an osteopathic medicine point of view, we need to review the mechanical characteristics of the brain. Sutherland himself, when citing the key points of his cranial model, wrote that to understand the mechanism of respiration of the cranial bones, it was necessary to include the brain [7]. The brain has a hysteretic behavior, that is, an elastic behavior that allows it to reconfigure its original morphology in the presence of internal or external deformations [26]. Mechanically, the brain is classified as a viscoelastic-porous body. It is a tissue full of fluids (80%-88%) and nonlinear solid material, constantly crossed by different rhythms of moving fluids and capable of distributing the tensions of the neurofluids in a way to recover or maintain its shape [26]. There are regional differences in the distribution of the mechanical stresses undergone but these tension vectors, once they reach the cortex, are equated [26]. White matter has greater stiffness (about one third more) than gray matter; white matter has a greater anisotropic characteristic and participates to a greater extent in the elasticity of the brain [27]. As we age, the brain acquires more stiffness, but preserves its ability to distribute the tensions that run through its solid-fluid structure [26]. The solid part is able to resist the force of fluids, increasing the hydrostatic pressure, in a hydromechanical continuum in constant motion and deformation [28]. The mechanical deformation forces that the nervous tissue undergoes from the passage of fluids and from the constant cranio-caudal and lateral-medial movement secondarily arising from the action of the heart and respiratory diaphragm are damped, precisely due to the intrinsic characteristic of the brain. The transmission of the mechanical forces presented to the outside will be distributed through the meninges with a decreased entity and speed [29]. We could say that the water-rich neurofluids make the brain organ like a tuning fork as all the notes of the body (solid, fluid, magnetic, electrical, and quantum information) are dispersed throughout the body and outside the body; the osteopath's hands read the sound, deciphering a symphony or a cacophony. The SRM is based on the movements of the brain mass induced by the myocardium and the diaphragm muscle, which, through the mechanical properties of the brain, neurofluids, meninges and the bone-suture complex, palpation detects cardiovascular and respiratory health. In addition, the health of the various layers from the brain to the osteopath's fingers is palpated. The contact between patient and operator, from a quantum physics point of view, creates a bi-directional meeting of magnetic and quantum information [30]. This meeting possibly influences water molecules, of which the brain is rich. For reasons not completely elucidated molecules of water may retain the memory of the substances with which they come into contact as a type of imprint, and influence the environment in which these water molecules persist [31-32]. Probably, this mechanism is one of the seeds in which self-healing matures.

The passive movement of the sacrum between the iliac bones

**Figure 3:**
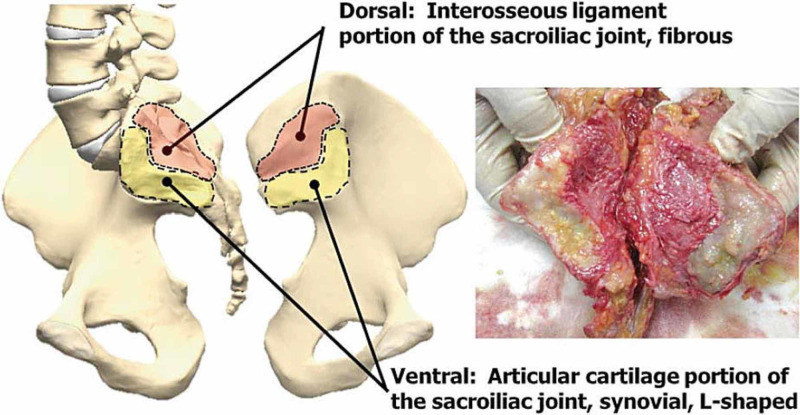
The figure illustrates the complexity of the SIJ and the different joint surfaces. SIJ, sacroiliac joint Thanks to Dr Kiapour A, Engineering Center for Orthopaedic Research Excellence (E-CORE), Departments of Bioengineering and Orthopedics, The University of Toledo, Toledo, Ohio, USA.

According to Sutherland's model, the sacral bone performs a rocking movement around a transverse axis (S2 and posterior to the sacral canal), called the respiratory axis; the movement occurs due to the inherent force of movement of the central nervous system, due to the fluctuations of the CSF and thanks to the cranial and spinal meninges [33]. There would be a sacral action of pulling upwards and releasing downwards, in a rhythmic and passive way; the rhythm per minute would correspond to the cranial rhythm found by palpation of the osteopath [33]. According to OCMM, during the flexion of the skull by the SBS, the sacral base is tractioned cranially/posteriorly (counter-nutation) as the sacral apex undergoes an anterior movement towards the pubis. During the extension of the skull, the sacral base is released in nutation (inferiorly/caudally), with its apex shifted towards the posterior [33]. There are no concrete studies to prove this theory. The sacroiliac joint (SIJ) is defined as an amphiarthrosis-diarthrosis (iliac bone and area S1 to S3 of the sacrum); the base of the sacrum (S1) and the last lumbar vertebra (L5) create a symphysis, while the respective joint facets form arthrodias [34]. The sacral bone ends its maturation at the age of 25-30, while the fusion of the sacral vertebrae ends at the age of 20 [34]. In 6% of the population of North America there is a sacralization, that is, the body of L5 merges with the base of the sacrum; there may be a partial fusion and a fusion that can involve the articular facets, with great articular morphological variability [34]. After the fourth decade of life, it is not uncommon to observe fusions between the apex of the sacrum and the coccygeal bone [34]. The joint area of the SIJ at the level of S3, with advancing age, becomes more sagittal, compared to the portions of S1 and S2; it is also possible to find small accessory joints of the SIJ [34]. The SIJ is innervated by the posterior branches of L5-S4; in the joint there are also encapsulated myelinated and unmyelinated fibers, which can be classified in the group of type IV and III fibers [34]. In an upright and sitting position, the angle between L5 and S1 becomes more acute, with a nutation of the sacrum, the posterior ligament complex is put in tension and the iliac bones tend to be translated dorsally [34]. The movement of the sacral bone, despite the musculature involved, is passive (Figure 3) [35].

**Figure 4:**
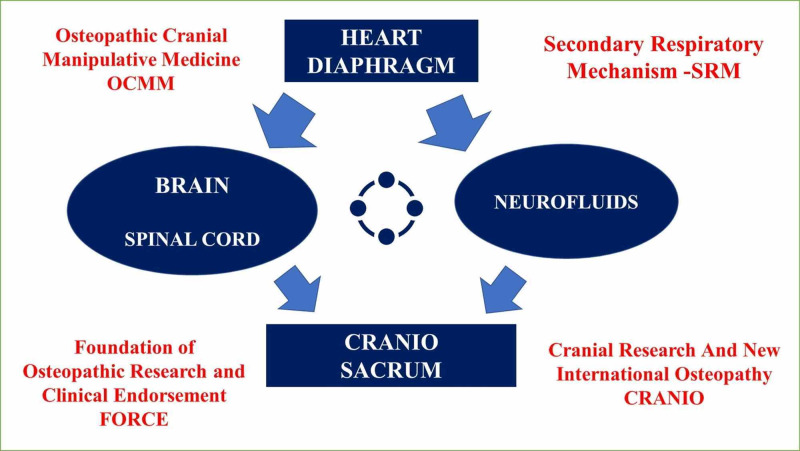
The scheme summarizes the concept of SRM. SRM, secondary respiratory mechanism

Sacral palpation of relevant anatomy by the osteopath is somewhat challenged by the current science. The sacral bone can move in all directions between the iliac bones, although the magnitude of the movement can rarely exceed 2 mm, with an average of 1.6 mm, and with a maximum of 2 degrees [34-35]. Each movement is the sum of several movements; for example, nutation and counter-nutation are the sum of a rotation and a translation [34]. During the gait cycle, in the oscillation phases where one leg bears the weight of the body, the SIJ is pulled downwards from the side of the free leg and undergoes a counter-nutation, with respect to the ileum; the ileum is lowered from the side of the swinging leg [36]. In the stance phase, the ileum on the side of foot contact is raised with respect to the sacrum, while the latter, passively performs a nutation [36]. In the oscillation phases the SIJ moves about 0.3 mm and about 0.6 mm in the support phases [36]. A study reveals that during the step, there must be an anticipatory movement of the sacral bone to improve the distribution of the body's loads [37]. When the patient is supine, the sacral base counter-nutation is the predominant position [34]. According to eminent authors, there is no valid manual test to adequately identify the movement of the sacrum with respect to the iliac bones, and no manual maneuver can alter the position of the sacral bone [34]. With manual approaches to SIJ, the position of the latter does not change, but neural responses are activated [decrease in the activity of the spinal motor neuron and decrease of Hoffman (H) reflex] [38-39]. What is the nature of the rhythms palpated by the osteopath at the level of the sacrum, compared to the identical rhythm manually recorded by the skull? We know that the sacrum is influenced not only by the movements of the legs, but also by the movements of the lumbar vertebrae and partly by the breath, which, by activating the pelvic floor musculature, slightly influences the sacral movement between the iliac bones [34-35]. Probably, to understand the nature of the cranio-sacral rhythm, we need to rethink the anatomy and functional characteristics of the spinal meninges and spinal neurofluids. The spinal dura mater at the lumbar and sacral level has a reduced thickness (103.74 ± 21.54 μm), compared to the cervical and thoracic areas; the inner layer has a greater number of collagen fibers than the outer layer, with a high capacity to withstand axial loads [40]. The dural sac ends at the level of S1-S2, the area where the transverse axis of sacral movements is identified [34, 41]. The dural sac is held in place by the meningovertebral ligaments, which are connected to the laminae and the yellow ligament [42]. In the sacral spinal meninges we find the arachnoid layer and the sub-arachnoid space with the same functional cerebral characteristics (as well as dural anisotropy) [40]. The pia mater, which covers the spinal cord, merges at the level of the filum terminale, passing beyond the medullary cone; it consists of collagen and reticular fibers [43]. The filum terminale covered by the pia mater is anchored to the coccyx, and the functional characteristics of the pia at this level, allow to maintain an adequate tension and elasticity status; the pia is covered at the level of the filum terminale by the subarachnoid space [43]. For better anchoring, the pial layer has denticulate ligaments, which create a more stable relationship between the medulla and the dural layer [43]. The lumbar and sacral spinal meninges have the same mechanical functions as the meninges of the skull. On an animal model, CSF at the sacral level has a higher speed than in the other spinal areas; it tends to accumulate at the sacral level, to be then absorbed through the sub-dural space by the lymphatic vessels [44]. In the sacral area we find a greater number of lymphatic vessels than in the other spinal areas; the remaining CSF travels through the epidural space (space between the dura mater and the flavum ligament), where there is no arachnoid barrier with the nerve exits [43-44]. At the level of L5-S2, the spinal cord widens in humans and this could coincide with the study on an animal model, where there is a greater collection of CSF and a greater presence of lymphatic vessels [45]. The medulla and the cauda equina, in addition to moving cranio-caudally for cardiac and respiratory stimulation, undergo oscillations of less than 1 mm, probably due to the oscillatory movement of the CSF [46]. These oscillations and movements of the diaphragm muscle and heartbeat do not coincide with the cranio-sacral rhythm. We can assume that at the sacral level (including bone) there are the same viscoelastic and mechanical conditions that allow these vital rhythms (heart and respiratory rate) to be cushioned and slowed down, as these variations in tension reach the surface (skin). We must also emphasize the fact that the SIJ joint itself has a high ability to dampen strong mechanical loads, and this joint congruence is another possible piece to understand the dichotomy between palpated rhythms and vital rhythms [36]. Body fluids and neurofluids give the shape and function of solid tissues, and allow the latter to maintain a salutogenic status [47]. We can hypothesize that when the osteopath palpates the skull and sacral bone, in addition to perceiving cardio-respiratory health (and other body areas), the clinician is able to palpate the SRM thanks to the neurofluids. Without the constant shifting of the neurofluids, there would be no perceptible rhythm. The osteopath is not a bone reshaper: the bones do not change position or shape as a result of manual stimuli, just as the cranial sutures do not diastasize. The manual stimuli that the osteopath can give with palpation of the skull and sacrum begin mechanistically from the epidermis. A gentle touch is able to activate afferents of myelinated and unmyelinated mechanoreceptors (Aβ, Aδ); this activation stimulates the release of opioid substances from the spinal cord, which will inhibit the nociceptive pathways thanks to the intervention of the parasympathetic system, the intervention of the somato-cardiac and respiratory reflex, with lowering of vital rhythms [48-49]. This mechanism is bi-directional (patient-operator) [49]. By acting on the lowering of the sympathetic system through the osteopathic manual approach, it is possible to assume that the osteopath's hands will affect the rhythms of neurofluids and health (Figure 4) [50].

## Conclusions

The second part of the article discussed the remaining components of OCMM, namely, the joint mobility of the bones of the skull, the SBS, the mechanical characteristics of the brain, and the movement of the sacrum between the iliac bones. The need to find another model for cranial osteopathic medicine that is able to more faithfully reflect the updated scientific notions was highlighted. We have proposed a new way of describing the mechanisms underlying OCMM; no longer PRM but SRM. To conclude, we highlighted the importance of neurofluids and the strategic function they play in the role of a salutogenic stimulus.
